# Supplementary material for: Heat adaptation of phage T7 under an extended genetic code
Source: Virus Evol. 2021 Dec 1;7(2):veab100. doi: 10.1093/ve/veab100 (PMC8923235; doi:10.1093/ve/veab100)
Supplement: veab100_Supp [file veab100_supp.zip › Sequencing_Analysis.zip!/Sequencing_Analysis/S3/evidence/JC_SIDE_2_53.html]

BRESEQ :: Evidence


| New junction evidence | | | | | | | | | | | |
| --- | --- | --- | --- | --- | --- | --- | --- | --- | --- | --- | --- |
|  | | seq id | position | reads (cov) | reads (cov) | score | skew | freq | annotation | gene | product |
| \* | ? | NC\_001604.1 | = 16604 | 551 (0.320) | 655 (0.390) | 233/590 | 1.5 | 51.6% | intergenic (–/–) | *–/–* | –/– |
|  | ? | NC\_001604.1 | 17028 = | 688 (0.410) |  |  |  |  | intergenic (–/–) | *–/–* | –/– |

Only 100 of 1405 total aligned reads displayed.  

`GACATTCTTGCGGTTGTGGTTGACGAAGATGTGCTTATTTTCACATGGGACGAAGTAAAAGGTAAGACATCCATGTGTGTCGGCAAGAGAAACAAAGGCATAAAACTATAGGAGAAATTATTATGGCTATGACAAAGAAATTTAAAGTGTCCTTCGACGTTACCGCAAAGATGTCGTCTGACGTTCAGGCAATCTTAGAGAAAGATATGCTGCATCTATGTAAGCAGGTCGGCTCAGGTGCGATTGTCCCCAATGGTAAACAGAAGGAAATGATTGTCCAGTTCCTGACACACGGTATGGAAGGATTGATGACATTCGTAGTACGTACATCATTTCGTGAGGCCATTAAGGACATGCACGAAGAGTATGCAGATAAGGACTCTTTCAAACAATCTCCTGCAACAGTACGGGAGGTGTTCTGATGTCTGACTACCTGAAAGTGCTGCAAGCAATCAAAAGTTGCCCTAAGACTTTCCAGTCCAACTATGTACGGAACAATGCGAGCCTCGTAGCGGAGGCCGCTTCCCGTGGTCACATCTCGTGCCTGACTACTAGTGGACGTAACGGTGGCGCTTGGGAAATCACTGCTTCCGGTACTCG  >  NC_001604.1/16729‑17328`  

                                                                                                                                                                                                                                                                                                           `|`                                                                                                                                                                                                                                                                                                              
  `cATTCTTGCGGTTGTGGTTGACGAAGATGTGCTTATTTTTACATGGGACGAAGTAAAAGGTAAGACATCCATGTGTGTCGGCAAGAGAAACAAAGGCATAAAACTATAGGAGAAATTATTATGGCGATGACAAAGAAATTTAAAGTGTCCTTCGACGTTACCGTAAAGATGTCGTCTGACGTTCAGGCAATCTTAGAGAAAGATATGCTGCATCTATGTAAGCAGGTCGGCTCAGGTGCGATTGTCCCCAATGGTAAACAGAAGGAAATGATTGTCCAGTTCCTGACACACGGTATGGAAg                                                                                                                                                                                                                                                                                                           <  1:148240/301‑1 (MQ=255)`  
  `aaTTCTTGCGGTTGTGGTTGACGAAGATGTGCTTATTTTCACATGGGACGAAGTAAAAGGTAAGACATCCATGTGTGTCGGCAAGAGAAACAAAGGCATAAAACTATAGGAGAAATTATTATGGCTATGAAAAAGAAATTTAAAGTGTCCTTCGACGTTACCGCAAAGATGTCGTCTGACGTTCAGGCAATCTTAGAGAAAGATATGCTGCATCTATGTAAGCAGGTCGGCTCAGGTGCGATTGTCCCCAATGGTAAACAGAAGGAAATGATTGTCCAGTTCCTGACACACGGTATGGAAg                                                                                                                                                                                                                                                                                                           <  1:276301/300‑1 (MQ=255)`  
    `ttCTTGCGGTTGTGGTTGACGAAGATGTGCTTATTTTCACATGGGACGAAGTAAAAGGTAAGTCATCCATGTGTGTCGGCAAGAGAAACAAAGGCATAAAACTATAGGAGAAATTATTATGGCTATGACAAAGAAATTTAAAGTGTCCTTCAACGTTACCGCAAAGATGTCGTCTGACGTTCAGGCAATCTTAGAGAAAGATATGCTGCATCTATGTAAGCAGGTCGGCTCAGGTGCGATTGTCCCCAATGGTAAACAGAAGGAAATGATTGTCCAGTTCCTGACACACGGTATGGAAGGa                                                                                                                                                                                                                                                                                                         >  1:256944/1‑301 (MQ=255)`  
           `agtccaagtgggctgccgtaccgaagagattggacaggtgggagttgggaccgcacaagaagcgatgcgctgggttggagaccactggaacttccggtggcttctggataacgaaagtaagatgtgtcctaattgggcgatttgccactgatacaggaggctactcatgaacgaaagacacttaacaggtgctgcttctgaaatgctagtagcctacaaatttaccaaagctgggtacactgtctatgaccctatgctgactcagagtaaagaggacttggttgtatgGAAGGATtgatga                                                                                                                                                                                                                                                                                                  <  1:150010‑M2/13‑1 (MQ=255)`  
                    `tGACGAAGATGTGCTTATTTTCACATGGGACGAAGTAAAAGGTAAGACATCCATGTGTGTCGGCAAGAGAAACAAAGGCATAAAACTATAGGAGAAATTATTATGGCTATGACAAAGAAATTTAAAGTGTCCTTCGACGTTACCGCAAAGATGTCGTCTGACGTTCAGGCAATCTTAGAGAAAGATATGCTGCATCTATGTAAGCAGGTCGGCTCAGGTGCGATTGTCCCCAATGGTAAACAGAAGGAAATGATTGTCCAGTTCCTGACACACGGTATGGAAGGATTGATGACATTCgtag                                                                                                                                                                                                                                                                                         <  1:110371/301‑1 (MQ=255)`  
                    `atgtgtataagagacagagattgctcaggtggtcattgagaccgcacaagaagcgatgcgttgggttggagaacactggaacttccggtgtcttctggataccgaaggtaagatgggtcctaatggggcgatttgccactgatacaggaggctactcatgaacgaaagacacttaacaggtgctgtttctgaaatgctagtagcctacaaatttaccaaagctgggtatactgtctattaccctatgctgactcagagtaaagaggacttggttgtatgGAAGGATTGATGACATtcgtct                                                                                                                                                                                                                                                                                         <  1:95447‑M2/22‑3 (MQ=255)`  
                       `ctgccgtaccgaagagattgctcaggtggtcattgagaccgcacaagaagcgatgcgctgggttggagaccactggaacttccggtgtcttctggataccgaaggtaagatgggtcctaattgggcgatttgccactgatacaggaggctactcatgaacgaaagacacttaacaggtgctgcttctgaaatgctagtagcctacaaatttaccaaagctgggtacactgtctattaccctatgctgactcagattaaagaggacttggttgtatgGAAGGATTGATGACATTCGTAgtac                                                                                                                                                                                                                                                                                      >  1:331624‑M2/277‑301 (MQ=255)`  
                         `aaGATGTGCTTATTTTCACATGGGACGAAGTAAAAGGTAAGACATCCATGTGTGTCGGCAAGAGAAACAAAGGCATAAAACTATAGGAGAAATTATTATGGCTATGACAAAGAAATTTAAAGTGTCCTTCGACGTTACCGCAAAGATGTCGTCTGACGTTCAGGCAATCTTAGAGAAAGATATGCTGCATCTATGTAAGCAGGTCGGCTCAGGTTCGATTGTCCCCAATGGTAAACAGAAGGAAATGATTGTCCAGTTCCTGACACACGGTATGGAAGGATTGATGACATTCGTAgtacgt                                                                                                                                                                                                                                                                                    >  1:236910/1‑301 (MQ=255)`  
                         `aaGATGTGCTTATTTTCACATGGGACGAAGTAAAAGGTAAGACATCCATGTGTGTCGGCAAGAGAAACAAAGGCATAAAACTATAGGAGAAATTATTATGGCTATGACAAAGAAATTTAAAGTGTCCTTCGACGTTACCGCAAAGATGTCGTCTGACGTTCAGGCAATCTTAGAGAAAGATATGCTGCATCTATGTAAGCAGGTCGGCTCAGGTGCGATTGTCCCCAATGGTAAACAGAAGGAAATGATTGTCCAGTTCCTGACACACGGTATGGAAGGATTGATGACATTCGTAgtactg                                                                                                                                                                                                                                                                                    >  1:138883/1‑299 (MQ=255)`  
                               `tgCTTATTTTCACATGGGACGAAGTAAAAGGTAAGACATCCATGTGTGTCGGCAAGAGAAACAAAGGCATAAAACTATAGGAGAAATTATTATGGCTATGACAAAGAAATTTAAAGTGTCCTTCGACGTTACCGCAAAGATGTCGTCTGACGTTCAGGCAATCTTAGAGAAAGATATGCTGCATCTATGTAAGCAGGTCGGCTCAGGTGCGATTGTCCCCAATGGTAAACAGAAGGAAATGATTGTCCAGTTCCTGACACACGGTATGGAAGGATTGATGACATTCGTAGTACGTAcatca                                                                                                                                                                                                                                                                              >  1:283519/1‑301 (MQ=255)`  
                                `gcgtATTTTCAAATGGGACGAAGTAAAAGGTAAGACATCCATGTGTGTCGGCAAGAGAAACAAAGGCATAAAACTATAGGAGAAATTATTATGGCTATGACAAAGAAATTTAAAGTGTCCTTCGACGTTACCGCAAAGATGTCGTCTGACGTTCAGGCACTCTTAGAGAAAGATATGCTGCATCTATGTAAGCAGGTCGGCTCAGGTGCGATTGTCCCCAATGGTAAACAGAAGGAAATGATTGTCCAGTTCCTGACACACGGTATGGAAGGATTGATGACATTCGTAGTACGTAcatcat                                                                                                                                                                                                                                                                             <  1:51865/298‑1 (MQ=255)`  
                                             `caggtggtcattgagaccgcacaagaagcgatgcgctgggttggagaccactggaatttccggtgtcttctggataccgaaggtaatatgggtcgtaattgggcgatttgccactgatacaggaggctactcatgaacgaaagacacttaacaggtgctgcttctgaaatgctagtagcctacaaatttaccaaagctgggtacactgcctattaccatatgctgacacagagtaaagaggacttggttgtatgGAAGGATTGATGACATTCGTAGTACGTACATCATTTCGTGAGGCAAt                                                                                                                                                                                                                                                                >  1:162749‑M2/255‑301 (MQ=255)`  
                                               `ggACGAAGTAAAAGGTAAGACATCCATGTGTGTCGGCAAGAGAAACAAAGGCATAAAACTATAGGAGAAATTATTATGGCTATGACAAAGAAATTTAAAGTGTCCTTCGACGTTACCGCAAAGATGTCGTCTGACGTTCAGGCAATCTTAGAGAAAGATATGCTGCATCTATGTAAGCAGGTCGGCTCAGGTGCGATTGTCCCCAATGGTAAACAGAAGGAAATGATTGTCCAGTTCCTGACACACGGTATGGAAGGATTGATGACATTCGTAGTACGTACATCATTTCGTGAGGCCATTa                                                                                                                                                                                                                                                              >  1:132127/1‑301 (MQ=255)`  
                                                  `ggtcattgagaccgcacaagaagcgatgcgctgggttggagaccactggaacttccggtgtcttctggataccgaaggtaagatgggtcctaattgggcgatttgccactgatacaggaggctactcatgaacgaaagacacttaacaggtgctgcttctgaaatgctagtagcctacaaatttaccaaagctgggtacactgtctattaccctatgctgactcagagtaaagaggacttggttgtatgGAAGGATTGATGACATTCGTAGTACGTACATCATTTCGTGAGGCCATTAAgg                                                                                                                                                                                                                                                           >  1:35510‑M2/250‑301 (MQ=255)`  
                                                    `aataaaaaTGAAAGACATCCATGGTTGTTGGCAAGATAAACAAAGGCATAAAACTATATGAGAAATTATTAGGGCTATGACAAAGAAATTTAAAGTGTCCTTCGACGTTACCGAAAAGATGTCGTCTGACGTTCAGGCAATCTTAGAGAAAGATATGCTGCATCTATGTAAGCAGGTCGGCTCAGGTGCGATTGTCCCCAATGGTAAACAGAAGGAAATGATTGTCCAGTTCCTGCCACACGGTATGGAAGGATTGATGACATTCGTAGTACGTACATCATTTCGTGAGGCCATTAAGGAc                                                                                                                                                                                                                                                         <  1:266989/297‑1 (MQ=255)`  
                                                    `aaGTAAAAGGGAAGACATACATGGGTGTCGGCAAGAGAAACAAAGGCATAAAACTATAGGAGAAATTATTATGGCTATGAAAAAGAAATTTAAAGTGTCCTTCGACGTTACCGCAAAGATGTCGTCTGACGTTCAGGCAATCTTAGAGAAAGATATGCTGCATCTATGTAAGCAGGTCGGCTCAGGTGCGATTGTCCCCAATGGTAAACAGAAGGAAATGATTGTCCAGTTCCTGACACACGGTATGGAAGGATTGATGACATTCGTAGTACGTACATCATTTCGTGAGGCCATTAAGGAc                                                                                                                                                                                                                                                         <  1:260111/301‑1 (MQ=255)`  
                                                      `gTAAAAGGTAAGACATCCATGTGTGTCGGCAAGAGAAACAAAGGCATAAAACTATAGGAGAAATTATTATGGCTATGACAAAGAAATTTAAAGTGTCCTTCGACGTTACCGCAAAGATGTCGTCTGACGTTCAGGCAATCTTAGAGAAAGATATGCTGCATCTATGTAAGCAGGTCGGCTCAGGTGCGATTGTCCCCAATGGTAAACAGAAGGAAATGATTGTCCAGTTCCTGACACACGGTATGGAAGGATTGATGACATTCGTAGTACGTACATCATTTCGTGAGGCCATTAAGGACAt                                                                                                                                                                                                                                                       >  1:135809/1‑301 (MQ=255)`  
                                                           `gaccgcacaagaagcgatgcgctgggttggagaccactggaacttccggtgtcttctggataccgaaggtaagatgggtcctaattgggcgatttgccactgatacaggaggctactcatgaacgaaagacacttaacaggtgctgcttctgaaatgctagtagcctacaaatttaccaaagctgggtacactgtctattaccctatgctgactcaaagtaaagaggacttggttgtatgGACGGATTGATGACATTCGTAGTACGTACATAATTTCGTGAGGCCATTAAGGacctgtctc                                                                                                                                                                                                                                                  >  1:239985‑M2/241‑297 (MQ=255)`  
                                                               `gacagaTCCATGTGTGTCGGCAAGAGAAACAAAGGCATAAAACTATAGGAGAAATTATTATGGCTATGACAAAGAAATTTAAAGTGTCCTTCGACGTTACCGCAAAGATGTCGTCTGACGTTCAGGCAATCTTAGAGAAAGATATGCTGCATCTATGTAAGCAGGTCGGCTCAGGTGCGATTGTCCCCAATGGTAAACAGAAGGAAATGATTGTCCAGTTCCTGACACACGGTATGGAAGGATTGATGACATTCGTAGTACGTACATCATTTCGTGAGGCCATTAAGGACATGCACGAaga                                                                                                                                                                                                                                              <  1:270859/296‑1 (MQ=255)`  
                                                                `tgggctcggagatgtggataagagacagccactggaacttccggtgtcttctggataccgaaggtaagatgggtcctaattgggcgatatgccactgatacaggaggatactcatgaacgaaagacacttaacaggtactgcttctgaaatgctagtagcctacaaatttaccaaagctgggtacactgtctattaccctatgctgactcagagtaaagaggacttggttgtatgGAAGGATTGATGACATTCGTAGTACGTACATCATTTCGTGAGGCCATTAAGGACATGCACGAagag                                                                                                                                                                                                                                             <  1:73870‑M2/66‑1 (MQ=255)`  
                                                                   `cATCCATGTGTGTCGGCAAGAGAAACAAAGGCATAAAACTATAGGAGAAATTATTATGGCTATGACAAAGAAATTTAAAGTGTCCTTCGACGTTACCGCAAAGATGTCGTCTGACGTTCAGGCAATCTTAGAGAAAGATATGCTGCATCTATGTAAGCAGGTCGGCTCAGGTGCGATTGTCCCCAATGGTAAACAGAAGGAAATGATTGTCCAGTTCCTGACACACGGTATGGAAGGATTGATGACATTCGTAGTACGTACATCATTTCGTGAGGCCATTAAGGACATGCACGAAGAGTAt                                                                                                                                                                                                                                          >  1:206135/1‑301 (MQ=255)`  
                                                                     `cccatggtggtCGGAAAAAGAAACAAAGGCATAAAACTATAGGAGAAATGATTATGGCTATGACAAAGAAATTTAAAGTGTCCTTCGACGTTACCGCAAAGATGTCGTCTGACGTTCAGGCAATCTTAGAGAAAGATATGCTGCATCTATGTAAGCAGGTCGGCTCAGGTGCGTTTGACCCCAATGGTAAACAGAAGGAAAGGATTGTCCAGTTCCTGACACACGGTATGGAAGGATTGATGACATTCGTAGTACGTACATCATTTCGTGAGGCCATTAAGGACATGCACGAAGAGTATGc                                                                                                                                                                                                                                        <  1:41916/292‑1 (MQ=255)`  
                                                                        `aTGTGTGTCGGAAAGAGAAACAAAGGCATAAAACTATAGGAGAAATTATTATGGTTATGACAAAGAAATTTAAAGTGTCCTTCGACGTTACCGCAAAGATGTCGTCTGACGTTCAGGCAATCTTAGAGAAAGATATGCTGCATCTATTTAAGCAGGTCGGCTCAGGTGCGATTGTCCCCAATGGTAAACAGAAGGAAATGATTGTCCAGTTCCTGACACACGGTATGGAAGGATTGATGACATTCGTAGTACGTACATCATTTCGTGAGGCCATTAAGGACATGCACGAAGAGTATGCAGa                                                                                                                                                                                                                                     <  1:209270/301‑1 (MQ=255)`  
                                                                            `gtgtCGGCAAGAGAAACAAAGGCATAAAACTATAGGAGAAATTATTATGGCTATGACAAAGAAATTTAAAGTGTCCTTCGACGTTACCGCAAAGATGTCGTCTGACGTTCAGGCAATCTTAGAGAAAGATATGCTGCATCTATGTAAGCAGGTCGGCTCAGGTGCGATTGTCCCCAATGGTAAACAGAAGGAAATGATTGTCCAGTTCCTGACACACGGTATGGAAGGATTGATGACATTCGTAGTACGTACATCATTTCGTGAGGCCATTAAGGACATGCACGAAGAGTATGCAGATAAg                                                                                                                                                                                                                                 >  1:182572/1‑301 (MQ=255)`  
                                                                             `gcgctgggttggagaccactggaacttccggtgtcttctggataccgaaggtaagatgggtcctaattgggcgatttgccactgatacaggaggctactcatgaacgaaagacacttaacaggtgctgcttctgaaatgctagtagcctacaaatttaccaaagctgggtacactgtctattaccctatgctgactcagagtaaagaggacttggttgtatgGAAGGATTGATGACATTCGTAGTACGTACATCATTTCGTGAGGCCATTAAGGACATGCACGAAGAGTAtgcagactgtc                                                                                                                                                                                                                                >  1:168914‑M2/223‑296 (MQ=255)`  
                                                                               `gcGGCAAGAGAAACAAAGGCATAAAACTAAAGGAGAAATTATTATAGATATGAAAAAGAAAGTGAAAGTGTCCGTCGACGTTACCACAAAGATGGCGTCTGACGGTCAGGCAATCTTAGAGAAAGATATGCTGCATCTATGTAAGCAGGTCGGCTCAGGTGCGATTGTCCCCAATGGTAAACAGAAGGAAATGATTGGCCAGTTCCTGACACACGGTATGGAAGGATTGATGACATTCGTAGTACGTACATCATTTCGTGAGGCCAGTAAGGACATGCACGAAGAGTATGAAGATAAGGAc                                                                                                                                                                                                                              <  1:176608/300‑1 (MQ=255)`  
                                                                                  `gggttggagaccactggaacttccggtgtcttctggataccgaaggtaagatgggtcctaattgggcgatttgccactgatacaggaggctactcatgaacgaaagacacttaacaggtgctgcttctgaaatgctagtagcctacaaatttaccaaagctgggtacactgtctattaccctatgctgactcagagtaaagaggacttggttgtatgGAAGGATTGATGACATTCGTAGTACGTACATCATTTCGTGAGGCCATTAAGGACATGCACGAAGAGTATGCAGATAAggacctt                                                                                                                                                                                                                           >  1:107439‑M2/218‑298 (MQ=255)`  
                                                                                  `gggttggagaccactggaacttccggtgtcttctggataccgaaggtaagatgggtcctaattgggcgatttgccactgatacaggaggctactcatgaacgaaagacacttaacaggtgctgcttctgaaatgctagtagcctacaaatttaccaaagctgggtacactgtctattaccctatgctgactcagagtaaagaggacttggttgtatgGAAGGATTGATGACATTCGTAGTACGTACATCATTTCGCGAGGCCATTAAGGACATGCACGAAGAGTATGCAGATAAgtacctg                                                                                                                                                                                                                           >  1:196452‑M2/218‑298 (MQ=255)`  
                                                                                    `gttggagaccactggaacttccggtgtcttctggataccgaaggtaagatgggtcctaattgggcgatttgccactgatacaggaggctactcatgaacgaaagacacttaacaggtgctgcttctgaaatgctagtatcctacaaatttaccaaagctgggtacactgtctattaccctatgctgactcagagtaaagaggacttggttgtatgGAAGGATTGATGACATTCGTAGTACGTACATCATTTCGTGAGGCCATTAAGGACATGCACGAAGAGTATGCAGATAAGGACTCttt                                                                                                                                                                                                                         <  1:192293‑M2/86‑1 (MQ=255)`  
                                                                                         `agagacaGGCAAAAAACTATAGGAAAAATTATTATGGCTAGGAAAAAGAAATTTAAAGTGTCCTTCGACGTTACCGCAAAGATGTCGTCTGACGTTCAGGCAATCTTAGAGAAAGATATGCTGCATCTATGTAAGCAGGTCGGCTCAGGTGCGATTGTCCCCAATGGTAAACAGAAGGAAATGATTGTCCAGTTCCTGACACACGGTATGGAAGGATTGATGACATTCGTAGTACGTACATCATTTCGTGAGGCCATTAAGGACATGCACGAAGAGTATGCAGATAAGGACTCTTTCAAAc                                                                                                                                                                                                                    <  1:344348/295‑1 (MQ=255)`  
                                                                                         `agagaaaGGCATAAAACTATAGGAGAAATTAGTATGGCTATGACAAAGAAATTTAAATTGTCCTTCGACGTTACCGCAAAGATGTCGTCTGACGTTCAGGCAATCTTAGAGAAAGATATGCTGCATCTATGTAAGCAGGTCGGCTCAGGTGCGATTGTCCCCAATGGTAAACAGAAGGAAATGATTGTCCAGTTCCTGACACACGGTATGGAAGGATTGATGACATTCGTAGTACGTACATCATTTCGTGAGGCCATTAAGGACATGCACGAAGAGTATGCAGATAAGGACTCTTTCAAAc                                                                                                                                                                                                                    <  1:334982/297‑1 (MQ=255)`  
                                                                                         `aaaCAAAGGAATAAAACTAGAGGAGAAATTATTATGGCTATGACAAAGAAATTTAAAGTGTCCTTCGACGTTACCGCAAAGATGTCGTCTAACGTTCAGGCAATCTTAGAGAAAGATATGCTGCATCTATGTAAGCAGGTCGGCTCAGGTGCGATTGTCCCCAATGGTAAACAGAAGGAAATGATTGTCCAGTTCCTGACACACGGTATGGAAGGATTGATGACATTCGTAGTACGTACATCATTTCGTGAGGCCATTAAGGACATGCACGAAGAGTATGCAGATAAGGACTCTTTCAAAc                                                                                                                                                                                                                    <  1:18988/301‑1 (MQ=255)`  
                                                                                               `ctggaacttccggtgtcttctggataccgaaggtaagatgggtcctaattgggcgatttgccactgatacaggaggctactcatgaacgaaagacacttaacaggtgctgcttctgaaatgctagtagcctacaaatttaccaaagctgggtacactgtctattaccctatgctgactcagagtaaagaggacttggttgtatgGAAGGATTGATGACATTCGTAGCACGTACATCATTTCGTGAGGCCATTAAGGACATGCACGAAGAGTATGCAGATAAGGCCTCTTTCCAAAAAtctc                                                                                                                                                                                                              >  1:198621‑M2/205‑301 (MQ=255)`  
                                                                                                   `aTAAAACTATAGGAGAAATTATGATGGCTATGACAAAGAAATTTAAAGTGTCCTTCGACGTTACCGCAAAGATGTCGTCTGACGTTCAGGCAATCTTAGAGAAAGATATGCTGCATCTATGTAAGCAGGTAGGCTCAGGTGCGATTGTCCCCAATGGTAAACAGAAGGAAATGATTGTCAAGTTCCTGACACACGGTATGGAAGGATTGATGACATTCGTAGTACGTACATCATTTCGTGAGGCCATTAAGGACATGCACGAAGAGTATGCAGATAAGGACTCTTTCAAACAATCTCCTGc                                                                                                                                                                                                          <  1:228732/301‑1 (MQ=255)`  
                                                                                                        `gcTATAGGAGAAATTATTATGGCTATGACAAAGAAATTTAAAGTGTCCTTCGACGTTACCGCAAAGATGTCGTCTGACGTTCAGGCAATCTTAGAGAAAGATATGCTGCATCTATGTAAGCAGGTCGGCTCAGGTGCGATTGTCCCCAATGGTAAACAGAAGGAAATGATTGTCCAGTTCCTGACACACGGTATGGAAGGATTGATGACATTCGTAGTACGTACATCATTTCGTGAGGCCATTAAGGACATGCACGAAGAGTATGCAGATAAGGACTCTTTCAAACAATCTCCTGCAACAg                                                                                                                                                                                                     <  1:67747/300‑1 (MQ=255)`  
                                                                                                        `aCTATAGGAGAAATTATTATGGCTATGACAAAGAAATTTAAAGTGTCCTTCGACGTTACCGCAAAGATGTCGTCTGACGTTCAGGCAATCTTAGAGAAAGATATGCTGCATCTATGTAAGCAGGTCGGCTCAGGTGCGATTGTCCCCAATGGTAAACAGAAGGAAATGATTGTCCAGTTCCTGACACACGGTATGGAAGGATTGATGACATTCGTAGTACGTACATCATTTCGTGAGGCCATTAAGGACATGCATGAAGAGTATGCAGATAAGGACTCTTTCAAACAATCTCCTGCAACAg                                                                                                                                                                                                     >  1:292888/1‑301 (MQ=255)`  
                                                                                                         `cTATAGGAGAAATTATTATGGCTATGACAAAGAAATTTAAAGTGTCCTTCGACGTTACCGCAAAGATGTCGTCTGACGTTCAGGCAATCTTAGAGAAAGATATGCTGCATCTATGTAAGCAGGTCGGCTCAGGTGCGATTGTCCCCAATGGTAAACAGAAGGAAATGATTGTCCAGTTCCTGACACACGGTATGGAAGGATTGATGAAATTCGTAGTACGTACATCATTTCGTGAGGCAATTAAGGACATGCACGAAGAGTATGCAGATAAGGAATCTTTCAAACCATCTCCTGCAACAGt                                                                                                                                                                                                    >  1:158820/1‑301 (MQ=255)`  
                                                                                                          `tataagagacagataccgaaggtaagatgggtcctaattgggcgatttgccactgatacaggaggctactcatgaacgaaagacacttaacaggtgctgcttctgaaatgctagtagcctacaaatttaccaaagctgggtacactgtctattaccctatgctgactcagagtaaagaggacttggttgtatgGAAGGATTGATGACATTCGTAGTACGTACATCATTTCGTGAGGCCATTAAGGACATGCACGAAGAGTATGTAGATAAGGACTCTTTCAAACAATCTCCTGCAACAGTa                                                                                                                                                                                                   <  1:233738‑M2/108‑1 (MQ=255)`  
                                                                                                          `ggtttcttctggataccgaaggtaagatgggtcctaattgggcgatttgccactgatacaggaggctactcatgaacgaaagacacttaacaggtgctgcttctgaaatgctagtagcctaccaatttaccaaagctgggtacactgtctattaccctatgctgactcagagtaaagaggacttggttgtatgGAAGGATTGATGACATTCGTAGTACGTACATCATTTCGTGAGGCCATTAAGGACATGCACGAAGAGTATGCAGATAAGGACTCTTTCAAAAAATCTCCTGCAACATTa                                                                                                                                                                                                   >  1:115591‑M2/194‑301 (MQ=255)`  
                                                                                                           `acagcttctggataccgaaggtaagatgggtcctaattgggcgatttgccactgatacaggaggctactcatgaacgaaagacacttaacaggtgctgcttctgaaatgctagtagcctacaaatttaccaaagctgggtacactgtctattaccctatgctgactcagagtaaagaggacttggttgtatgGAAGGATTGATGACATTCGTAGTACGTACATCATTTCGTGAGGCCATTAAGGACATGCACGAAGAGTATGCAGATAAGGACTCTTTCAAACAATCTCCTGCAACAGTAc                                                                                                                                                                                                  <  1:203753‑M2/109‑1 (MQ=255)`  
                                                                                                             `gtcttctggataccgaaggtaagatgggtcctaattgggcgatttgccactgatacaggaggctactcatgaacgaaagacacttaacaggtgctgcttctgaaatgctagtagcctacaaatttaccaaagctgggtacactgtctattaccctatgctgactcagagtaaagaggacttggttgtatgGAAGGATTGATGACATTCGTAGTACGTACATCATTTCGTGAGGCCATTAAGGACATGCACGAAGAGTATGCAGATAAGGACTCTTTCAAACAATCTCCTGcaacagctgtc                                                                                                                                                                                                >  1:289262‑M2/191‑296 (MQ=255)`  
                                                                                                               `cttctggataccgaaggtaagatgggtcctaattgggcgatttgccactgatacaggaggctactcatgaacgaaagacacttaacaggtgctgcttctgaaatgctagtagcctacaaatttaccaaagctgggtacactgtctattaccctatgctgactcagagtaaagaggacttggttgtatgGAAGGATTGATGACATTCGTAGTACGTACATCATTTCGTGAGGCCATTAAGGACATGCACGAAGAGTATGCAGATAAGGACTCTTTCAAACAATCTCCTGCAACAGTACGGGa                                                                                                                                                                                              >  1:153541‑M2/189‑301 (MQ=255)`  
                                                                                                                 `aaaaTTATTATGGCTATGAAAAAGAAATTTAAAGTGTCCTTCGACGTTACCGCAAAGATGTCGTCTGACGTTCAGGCAATCTTAGAGAAAGATATGCTGCATCTATGTAAGCAGGTCGGCTCAGGTGCGATTGTCCCCAATGGTAAACAGAAGGAAATGATTGTCCAGTTGCTGACACACGGTATGGAAGGATTGATGACATTCGTAGTACGTACATCATTTCGTGAGGCCATTAAGGACATGCACGAAGAGTATGCAGATAAGGACTCTTTCAAACAATCTCCTGCAACAGTACGGGAgg                                                                                                                                                                                            <  1:341802/300‑1 (MQ=255)`  
                                                                                                                      `tattatGGCGATGACAAAGAAATTTAAAGTGTCCGTCGACGTTACCGCAAAGATGTCGTCTGACGTTCAGGCAATCTTAGAGAAAGATATGCTTCATCTATGTAAGCAGGTCGGCTCAGGTGCGATTGTCCCCAATGGTAAACAGAAGGAAATGATTGTCCAGTTCCTGACACACGGTATGGAAGGATTGATGACATTCGTAGTACGTACATCATTTCGTGAGGCCATTAAGGACATGCACGAAGAGTATGCAGATAAGGACTCTTTCAAACAATCTCCTGCAACAGTACGGGAGGTGTTc                                                                                                                                                                                       <  1:326086/301‑1 (MQ=255)`  
                                                                                                                         `tatGGCTATGACAAAGACATTTAAAGTGTCCTTCGACGTTACCGCAAAGATGTCGTCTGAAGTTCAGGCAATCTTAGAGAAAGATATGCTGCATCTATGTAAGCAGGTCGGCTCAGGTGCGATTGTCCCCAATGGTAAACAGAAGGAAATGATTGTCCAGTTCCTGACACACGGTATGGAAGGATTGATGACATTCGTAGTACGTACATCATTTCGTGAGGCCATTAAGGACATGCACGAAGAGTATGCAGATAAGGACTCTTTCAAACAATCTCCTGCAACAGTACGGGAGGTGTTCTGa                                                                                                                                                                                    <  1:63020/301‑1 (MQ=255)`  
                                                                                                                            `ggCTATGACAAAGAAATTTAAAGTGTCCTTCGACGTTACCGCAAAGATGTCGTCTGACGTTCAGGCAATCTTAGAGAAAGATATGCTGCATCTATGTAAGCAGGTCGGCTCAGGTGCGATTGTCCCCAATGGTAAACAGAAGGAAATGATTGTCCAGTTCCTGACACACGGTATGGAAGGATTGATGACATTCGTAGTACGTACATCATTTCGTTAGGCCATTAAGGACATGCACGAAGAGTATGCAGATAAGGACTCTTTCAAACAATCTCCTGCAACCGTACGGGAGGTGTTCTGATGt                                                                                                                                                                                 >  1:44218/1‑301 (MQ=255)`  
                                                                                                                                       `ggtcctaattgggcgatttgccactgatacaggaggctactcatgaacgaaagacacttaacaggtgctgcttctgaaatgctagtatcctacaaatttaccaaagctgggtacactgtctattaccctatgctgactcagagtaaagaggacttggttgtatgGAAGGATTGATGACATTCGTAGTACGTACATCATTTCGTGAGGCCATTAAGGACATGCACGAAGAGTATGCAGATAAGGACTCTTTCAAACAATCTCCTGCAACAGTACGGGAGGTGTTCTGATGTCTGACTACCTg                                                                                                                                                                      >  1:281443‑M2/165‑301 (MQ=255)`  
                                                                                                                                          `aaTTTAAAGTGTCCTTCGACGTTACCGCAAAGATGTCGTCTGACGTTCAGGCAATCTTAGAGAAAGATATGCTTCATCTATGTAAGCAGGTCGGCTCATGTGCGATTGTCCCCAATGTTAAACAGAAGGAAATGATTGTCCTGTTCCTGACACCCGGTATTGAAGGATTTATCACATTCGTAGTACGTACATCATTTCGTGAGTCCATTAAGGACATGCACGAAGCGTATGCAGATAAGGACTCTTTCAAACAATCTCCTGCAACAGTACGTGAGCTGTCCTGATGTCTGACTACCtttaa                                                                                                                                                                   >  1:112940/1‑297 (MQ=255)`  
                                                                                                                                           `ttaattgggcgatttgcaactgatacaggaggctaatcatgaacgaaagacacttaacaggtgctgcttctgaaatgccagtagcctacaaattaaccaaagctgggtacactgtctattaccctatgctgactcagagtaaagaggacttggttgtatgGAAGGATTGATGACATTCGTAGTACGTACATCATTTCGTGAGGCCATTAAGGACATGCACGAAGAGTATGCAGATAAGGACTCTTGCAAACAATCTCCTGCAACAGTACGGGAGGTGTTCTGATGTCTGACTACCTAAAAg                                                                                                                                                                  <  1:155781‑M2/141‑1 (MQ=255)`  
                                                                                                                                                              `ctgatacaggaggctactcatgaacgaaagacacttaacaggtgctgcttctgaaatgctagtagcctacaaatttaccaaagctgggtacactgtctattaccctatgctgactcagagtaaagaggacttggttgtatgGAAGGATTGATGACATTCGTAGTACGTACATCATTTCGTGAGGCCATTAAGGACATGCACGAAGAGTATGCAGATAAGGACTCTTTCAAACAATCTCCTGCAACAGTACGGGAGGTGTTCTGATGTCTGACTACCTGAAAGTGCTGCAAGCAATCAAAAg                                                                                                                                               >  1:12359‑M2/142‑301 (MQ=255)`  
                                                                                                                                                                 `atacaggaggctactcatgaacgaaagacacttaacaggtgctgcttctgaaatgctagtagcctacaaatttaccaaagctgggtacactgtctattaccctatgctgactcagagtaaagaggacttggttgtatgGAAGGATTGATGACATTCGTAGTACGTACATCATTTCGTGAGGCCATTAAGGACATGCACGAAGAGTATGCAGATAAGGACTCTTTCAAACAATCTCCTGCAACAGTACGGGAG‑‑GTTCTGATGTCTGACTACCTGAAAGTGCTGCAAGCAATCAAAAGTTGcc                                                                                                                                          >  1:59165‑M2/139‑301 (MQ=255)`  
                                                                                                                                                                   `cGCAAAGATGTCGTCTGACGTTCAGGCAATCTTAGAGAAAGATATGCTGCATCTATGTAAGCAGGTCGGCTCAGGTGCGATTGTCCCCAATGGTAAACAGAAGGAAATGATTGTCAAGTTCCTGACACACGGTATGGAAGGATTGATGACATTCGTAGTACGTACATCATTTCGTGAGGCCATTAAGGACATGCACGAAGAGTATGCAGATAAGGACTCTTTCAAACAATCTCCTGCAACAGTACGGGAGGTGTTCTGATGTCTGACTACCTGAAAGTGCTGCAAGCAATCAAAAGTTGcc                                                                                                                                          <  1:273592/301‑1 (MQ=255)`  
                                                                                                                                                                    `aaagaggctaatcatgaacgaaagacacttaacaggtgctgcttctgaaatgctagtagcctacaaatttaccaaagctgggtacactgtctattaccctatactgactcagagtaaagaggacttggttgtatgGAAGGATTGATGACATTCGTAGTACGTACATCATTTCGTGAGGCCATTAAGGACATGCACGAAGAGTATGCAGATAAGGACTCTTTCAAACAATCTCCTGCAACAGTACGGGAGGTGTTCTGATGTCTGACTACCTGAAAGTGCTGCAAGCAATCAAAAGTTGccc                                                                                                                                         <  1:80976‑M2/166‑1 (MQ=255)`  
                                                                                                                                                                          `ggagatgtgtataagagacagcttaacaggtgctgcttctgaaatgctagtagcctacaaatttaccaaagctgggtacactgtctattaccctatgctgactcagagtaaagaggacttggttgtatgGAAGGATTGATGACATTCGTAGTACGTACATCATTTCGTGAGGCCATTAAGGACATGCACGAAGAGTATGCAGATAAGGACTCTTTCAAACAATCTCCTGCAACAGTACGGGAGGTGTTCTGATGTCTGACTACCTGAAAGTGCTGCAAGCAATCAAAAGTTGCCCTAAGAc                                                                                                                                   <  1:125624‑M2/172‑1 (MQ=255)`  
                                                                                                                                                                          `gctactcatgaacgaaagacacttaacaggtgctgcttctgaaatgctagtagcctacaaatttaccaaagctgggtacactgtctattaccctatgctgactcagagtaaagaggacttggttgtatgGAAGGATTGATGACATTCGTAGTACGTACATCATTTCGTGAGGCCATTAAGGACATGCACGAAGAGTATGCAGATAAGGACTCTTTCAAACAATCTCCTGCAACAGTACGGGAGGTGTTCTGATGTCTGACTACCTGAAAGTGCTGCAAGCAATCAAAAGTTGCCCTAAGAc                                                                                                                                   <  1:318706‑M2/172‑1 (MQ=255)`  
                                                                                                                                                                                    `aacgaaagacacttaacaggtgctgcttctgaaatgatagtagcctacaaatttaccaaagctgggtacactgtctattaccctatgctgactcagagtaaagaggacttggttgtatgGAAGGATCGATGACATTCGTAGTACGTACATCATTTCGTGAGGCCATTAAGGGCATGCACGAAGAGTATGCAGATAAGGACTCTTTCAAACAATATCCTGCAACAGTACGGGAGGTGTTCTGATGTCTGACTACCTGAAAGTGCTGCAAGCAATCAAAAGTTGCCCTAAGACTTTCCAGTcc                                                                                                                         <  1:38242‑M2/182‑1 (MQ=255)`  
                                                                                                                                                                                     `cGTTCAGGCAATCTTAGAGAAAGATATGCTGCATCTATGTAAGCAGGTCGGCTCAGGTGCGATTGTCCCCAATGGTAAACAGAAGGAAATGATTGTCCAGTTCCTGACACACGGTATGGAAGGATTGATGACATTCGTAGTACGTACATCATTTCGTGAGGCCATTAAGGACATGCACGAAGAGTATGCAGATAAGGACTCTTTCACACAATCTCCTGCACCAGTACGGGAGGTGTTCTGATGTATGACTACCTGAAAGTGCTGCAAGCAATCAAAAGTTGCCCTAAGACTTTCCAGTCCa                                                                                                                        >  1:287086/1‑301 (MQ=255)`  
                                                                                                                                                                                     `cGTTCAGGCAATCTTAGAGAAAGATATGCTGCATCTATGTAAGCAGGTCGGCTCAGGTGCGATTGTCCCCAATGGTAAACAGAAGGAAATGATTGTCCAGTTCCTGACACACGGTATGGAAGGATTGATGACATTCGTAGTACGTACATCATTTCGTGAGGCCATTAAGGACATGCACGAAGAGTATGCAGATAAGGACTCTTTCAAACAATCTCCTGCAACAGTACGGGAGGTGTTCTGATGTCTGACTACCTGAAAGTGCTGCAAGCAATCAAAAGTTGCCCTAAGACTTTCCAGTCCa                                                                                                                        >  1:78110/1‑301 (MQ=255)`  
                                                                                                                                                                                             `cacttaacaggtgctgcttctgaaatgctagtagcctacaaatttaccaaagctgggtacactgtctattaccctatgctgactcagagtaaagaggacttggttgtatgGAAGGATTGATGACATTCGTAGTACGTACATCATTTCGTGAGGCCATTAAGGACATGCACGAAGAGTATGCAGATAAGGACTCTTTCAAACAATCTCCTGCAACAGTACGGGAGGTGTTCTGATGTCTGACTACCTGAAAGTGCTGCAAGCAATCAAAAGTTGCCCTAAGAATTTCCAGTCCAACTATGTa                                                                                                                >  1:329251‑M2/111‑301 (MQ=255)`  
                                                                                                                                                                                               `cttaacaggtgctgcttctgaaatgctagtagcctacaaatttaccaaagctgggtacactgtctattaccctatgctgactcagagtaaagaggacttggttgtatgGAAGGATTGATGACATTCGTAGTACGTACATCATTTCGTGAGGCCATTAAGGACATGCACGAAGAGTATGCAGATAAGGACTCTTTCAAACAATCTCCTGCAACAGTACGGGAGGTGTTCTGATGTCTGACTACCTGAAAGTGCTGCAAGCAATCAAAAGTTGCCCTAAGACTTTCCAGTCCAACTATGTACg                                                                                                              >  1:165467‑M2/109‑301 (MQ=255)`  
                                                                                                                                                                                               `aTCTTAGAGAAAGATATGCTGCATCTATGTAAGCAGGTCGGCTCAGGTGCGATTGTCCCCAATGGTAAACAGAAGGAAATGATTGTCCAGTTCCTGACACACGGTATGGAAGGATTGATGACATTCGTAGTACGTACATCATTTCGTGAGGCCATTAAGGACATGCACGAAGAGTATGCAGATAAGGACTCTTTCAAACAATCTCCTGCAACAGTACGGGAGGTGTTCTGATGTCTGACTACCTGAAAGTGCTGCAAGCAATCAAAAGTTGCCCTAAGACTTTCCAGTCCAACTATGTAcc                                                                                                              >  1:133904/1‑300 (MQ=255)`  
                                                                                                                                                                                                 `gaggtgtataagagacaggcATCTATGTAAGCAGGTCGGCTCAGGTGCGATTGTCCCAAATGGTAAACAGAAGGAAATGATTGTCCAGTTCCTGACACACGGTATGGAAGGATTGATGACATTCGTAGTACGTACATCATTTCGTGAGGCCATTAAGGACATGCACGAAGAGTATGCAGATAAGGACTCTTTCAAACAAACTCCTGCAACAGTACGGGAGGTGTTCTGATGTCTGACTACCTGAAAGTGCTGCAAGCAATCAAAAGTTGCCCTAAGACTTTCCAGTCCAACTATGTACGGa                                                                                                            <  1:53573/283‑1 (MQ=255)`  
                                                                                                                                                                                                   `acaggtgctgcttctgaaatgctagtagcctacaaatttaccaaagctgggtacactgtctattaccctatgctgactcagagtaaagaggacttggttgtatgGAAGGATTGATGACATTCGTAGTACGTACATCATTTCGTGAGGCCATTAAGGACATGCACGAAGAGTATGCAGATAAGGACTCTTTCAAACAATCTCCTGCAACAGTACGGGAGGTGTTCTGATGTCTGACTACCTGAAAGTGCTGCAAGCAATCAAAAGTTGCCCTAAGACTTTCCAGTCCAACTATGTACGGAAc                                                                                                          <  1:186833‑M2/197‑1 (MQ=255)`  
                                                                                                                                                                                                        `ataagagcaaggcATCTATGTAAGCATGTCCGCTCAGGTGCGATTGTCCCCAAGGGTAAACAGAATGAAAGGATTGTCCAGTTCCTGACACACGGTATGGAAGGATTGATGACATTCGTAGTACGTACATCATTTCGTGAGGCCATTAAGGACATGCACGAAGAGTATGCAGATAAGGACTCATTCAAACAATCTCCTGTAACAGTACGGGAGGTGTTCTGATGTCTGACTACCTGAAAGTGCTGCAAGCAATCAAAAGTTGCCCTAAGACTTTCCAGTCCAACTATGTACGGAACAATGc                                                                                                     <  1:213662/290‑1 (MQ=255)`  
                                                                                                                                                                                                        `aaaGATATTCTTCATCTATGTAAGCAGGTCGGCGCAGGTGCGATTGTACCCAATGGGAAACAGAAGGAAATGATTGTCCAGTTCCTGACACACGGTATGGAAGGATTGATGACATTCGTAGTACGTACATCATTTCGTGAGGCCATTAAGGACATGCACGAAGAGTATGCAGATAAGGACTCTTTCAAACAATCTCCTGCAACAGTACGGGATGTGTTCTGATGTCTGACTACCTGAAAGTGCTGCAAGCAATCAAAAGTTGCCCTAAGACTTTCCAGTCCAACTATGTACGGAACAATGc                                                                                                     <  1:21527/301‑1 (MQ=255)`  
                                                                                                                                                                                                           `gATATGCTGCATCTATGTAAGCAGGTCGGCTCAGGTGCGATTGTCCCCAATGGTAAACAGAAGGTAATGATTGTCCAGTTCCTGACACACGGTATGGAAGGATTGATGACATTCGTAGTACGTACATCATTTCGTGAGGCCATTAAGGACATGCACGAAGAGTATGCAGATAAGGACTCTTTCAAACAATCTCCTGCAACAGTACGGGAGGTGTTCTGATGTCTGACTACCTGAAAGTGCTGCAAGCAATCAAAAGTTGACCTAAGACTTTCCAGTCCAACTATGTACGGAACAAGGCGAg                                                                                                  >  1:55952/1‑301 (MQ=255)`  
                                                                                                                                                                                                                    `aatgcaagtagcctacaaatttaccaaagctgggtacactgtctattaccctatgctgactcagagtaaagaggacttggttgtatgGAAGGATTGATGACATTCGTAGTACGTACATCATGTCGTGAGGCCATTAGGGAAATGCACGACGAGTATACAGATAAGGACTCATTAAAGCAATATCATGCAACAGTATGGGCGGTGTTCTGATGTCAGACTACCTGAAAGTACTGAAAGCAATCACAAGTGGACATAAGACTTTCAAGTCAAACTATGTCGGGAAAAATGCGAGCTTCGGagg                                                                                         >  1:248673‑M2/88‑300 (MQ=255)`  
                                                                                                                                                                                                                     `atgctagtagcctacaaatttacaaaagctgggtacactgtctattaccctatgctgaatcagagtaaagaggacttggttgtatgGAAGGATTGATGACATTCGTAGTACGTACATCATTTCGTGAGGCCATTAAGGACATGCACGAAGAGTATGCAGATAAGGACTCTTTCAAACAATCTCCTGCAACAGTACGGGAGGTGTTCTGATGTCTGACGACCTGAAAGTGCTGCAAGCAATCAAAAGTTGCCCTAAGACTTTCCAGTCCAACTATGTACGGAACAATGCGAGCCTCGTAGCg                                                                                        <  1:20117‑M2/215‑1 (MQ=255)`  
                                                                                                                                                                                                                      `atgtgtataagagacaggCTCAGGTGCGATTGTCCCCAATGGTAAACAGAAGGAAATGATTGTCCAGTTCCTGACACACGGTATGGAAGGATTGATGACATTCGTAGTACGTACATCATTTCGTGAGGCCATTAAGGACATGCACGAAGAGTATGCAGATAAGGACTCTTTCAAACAATCTCCTGCAACAGTACGGGAGGTGTTCTGATGTCTGACTACCTGAAAGTGCTGCAAGCAATCAAAAGTTGCCCTAAGACTTTCCAGTCCAACTATGTACGGAACAATGCGAGCCTCGTAGCgg                                                                                       <  1:87948/285‑1 (MQ=255)`  
                                                                                                                                                                                                                       `cTATGTAAGCAGGTCGGCTCAGGTGCGATTGTCCCCAATGGTAAACAGAAGGAAATGATAGTCCAGTTCCTGACACACGGTATGGAAGGATTGATGACATTCGTAGTACGTACATCATTTCGTGAGGCCATTAAGGACATGCACGAAGAGTATGCAGATAAGGACTCTTTCAAACAATCTCCTGCAACAGTACGGGAGGTGTTCTGATGTCTGACTACCTGAAAGTGCTGCAAGCAATCAAAAGTTGCCCTAAGACTTTCCAGTCCAACTATGTACGGAAcctgtctcttatacacatctc                                                                                      >  1:218468/1‑281 (MQ=255)`  
                                                                                                                                                                                                                        `ctagtagcctacaaatttaccaaagctgggtacactgtctattaccctatgctgactcagagtaaagaggacttggttgtatgGAAGGATTGATGACATTCGTAGTACGTACATCATTTCGTGAGGCCATTAAGGACATGCACGAAGAGTATGCAGATAAGGACTCTTTCAAACAATCTCCTGCAACAGTACGGGAGGTGTTCTGATGTCTGACTACCTGAAAGTGCTGCAAGCAATCAAAAGTTGCCCTAAGActttccagtccaactatgtacggactgtctcttatacacatctccga                                                                                     >  1:91080‑M2/84‑278 (MQ=255)`  
                                                                                                                                                                                                                          `agtagcctacaaatttaccaaagctgggtacactgtctattaccctatgctgactcagagtaaagaggacttggttgtatgGAAGGATTGATGACATTCGTAGTACGTACATCATTTCGTGAGGCCATTAAGGACATGCACGAAGAGTATGCAGATAAGGACTCTTTCAAACAATCTCCTGCAACAGTACGGGAGGTGTTCTAATGTCTGACTACCTGAAAGTGCTGCAAGCAATCAAAAGTTGCCCTAAGACTTTCCAGTCCAACTATGTACGGAACAATGCGAGCCTCGTAGCGGAGGc                                                                                   <  1:69875‑M2/220‑1 (MQ=255)`  
                                                                                                                                                                                                                                 `tacaaatttaccaaagctgggtacactgtctattaccctatgctgactcagagtaaagaggacttggttgtatgGAAGGATTGATGACATTCGTAGTACGTACATCATTTCGTGAGGCCATTAAGGACATGCACGAAGAGTATGCAAATAAGGACTCTTTCAAACAAGCTCCTGCAACAGTACGGGAGGTGTTCTGATGTCTGACTACCTGAAAGTGCTGCAAGCAATCAAAAGTTGCCCTAAGACTTTCCAGTCCAACTATGTACGGAACAATGCGAGCCTCGTAGCGGAGGCCGCTTcc                                                                            <  1:316055‑M2/227‑1 (MQ=255)`  
                                                                                                                                                                                                                                  `acacttaacAGGTGCGATTGTCCCCAATGGTAAACAGAAGGAAATGATTGTCCAGTTCCTGACACACGGTATGGAAGGATTGATGACATTCGTAGTACGTACATCATTTCGTGAGGCCATTAAGGACATGCACGAAGAGTATGCAGATAAGGACTCTTTCAAACAATCTCCTGCAACAGTACGGGAGGTGTTCTGATGTCTGACTACCTGAAAGTGCTGCAAGCAATCAAAAGTTGCCCTAAGACTTTCCAGTCCAACTATGTACGGAACAATGCGAGCCTCGTAGCGGAGGCCGCTTccc                                                                           >  1:119339/9‑301 (MQ=255)`  
                                                                                                                                                                                                                                   `cacttaacAGGTGCGATTGTCCCCAATGGTAAACAGAAGGAAATGATTGTCCAGTTCCTGACACACGGTATGGAAGGATTGATGACATTCGTAGTACGTACATCATTTCGTGAGGCCATTAAGGACATGCACGAAGAGTATGCAGATAAGGACTCTTTCAAACAATCTCCTGCAACAGTACGGGAGGTGTTCTGATGTCTGACTACCTGAAAGTGCTGCAAGCAATCAAAAGTTGCCCTAAGACTTTCCAGTCCAACTATGTACGGAACAATGCGAGCCTCGTAGCGGAGGCCGCTTcccc                                                                          >  1:173029/8‑300 (MQ=255)`  
                                                                                                                                                                                                                                              `aagctgggtacactgtcgattaccctatgctgactcagagtaaagaggacttggttgtatgGAAGGATTGATGACATTCGTAGTACGTACATCATTTCGTGAGGCCATTAAGGACATGCACGAAGAGTATGCAGATAAGGACTCTTTCAAACAATCTCCTGCAACAGTACGGGAGGTGTTCTGATGTCTGACTACCTGAAAGTGCTGCAAGCAATCAAAAGTTGCCCTAAGACTTTCCAGTCCAACTATGTACGGAACAATGCGAGCCTCGTAGCGGAGGCCGCTTCCCGTGGTCACAtct                                                               <  1:65240‑M2/240‑1 (MQ=255)`  
                                                                                                                                                                                                                                                  `aTTGTCCCCAATGGTAAACAGAAGGAAATGATTGTCCAGTTCCTGACACACGGTATGGAAGGATTGATGACATTCGTAGTACGTACATCATTTCGTGAGGCCATTAAGGACATGCACGAAGAGTATGCAGATAAGGACTCTTTCAAACAATCTCCTGCAACAGTACGGGAGGTGTTCTGATGTCTGACTACCTGAAAGTGCTGCAAGCAATCAAAAGTTGCCCTAAGACTTTCCAGTCCAACTATGTACGGAACAATGCGAGCCTCGTAGCGGAGGCCGCTTCCCGTGGTCACATCTCGTg                                                           <  1:311567/301‑1 (MQ=255)`  
                                                                                                                                                                                                                                                    `tGTCCCCAATGGTAAACAGAAGGAAATGATTGTCCAGTTCCTGACACACGGTATGGAAGGATTGATGACATTCGTAGTACGTACATCATTTCGTGAGGCCATTAAGGACATGCACGAAGAGTATGCAGATAAGGACTCTTTCAAACAATCTCCTGCAACAGTATGGGAGGTGTTCTGATGTCTGACTACCTGAAAGTGCTGCAAGCAATCAAAAGTTGCCCTAAGACTTTCCAGTCCAACTATGTACGGAACAATGCGAGCCTCGTAGCGGAGGCCGCTTCCCGTGGTCACATCTCGTGcc                                                         <  1:144468/301‑1 (MQ=255)`  
                                                                                                                                                                                                                                                     `gtacactgtctattaccctatgctgactcagagtaaagaggacttggttgtatgGAAGGATTGATGACATTCGTAGTACGTACATCATTTCGTGAGGCCATTAAGGACATGCACGCAGAGTATGCAGATAAGGACTCTTTCAAACAATCTCCTGCAACAGTACGGGAGGTGTTCTGATGTCTGACTACCTGAAAGTGCTGCAAGCAATCAAAAGTTGCCCTAAGACTTTCCAGTCCAACTATGTACGGAACAATGCGAGCCTCGTAGCGGAGGCCGCTTCCCGTGGTCACATCTCGTGCCt                                                        >  1:48135‑M2/55‑301 (MQ=255)`  
                                                                                                                                                                                                                                                     `gTCCCCAATGGTAAACAGAAGGAAATGATTGTCCAGTTCCTGACACACGGTATGGAAGGATTGATGACATTCGTAGTACGTACATCATTTCGTGAGGCCATTAAGGACATGCACGAAGAGTATGCAGATAAGGACTCTTTCAAACAATCTCATGCAACAGTACGGGAGGTGTTCTGATGTCTGACTACCTGAAAGTGCTGCAAGCAAACAAAAGTTGCCCTAAGACTTGCCAGTCCAACTATGTACGGAACAATGCGAGCCTCGTAGCAGAGGCCACTTCCCGTGGTCACATCTCGTGCCt                                                        >  1:321661/1‑301 (MQ=255)`  
                                                                                                                                                                                                                                                              `ggTAAACAGAAGGAAATGATTGTCCAGTTCCTGACACATGGTATGGAAGGATTGATGACATTCGTAGTACGTACATCATTTCGTGAGGCCATTAAGGACATGCACGAAGAGTATGCAGATAAGGACTCTTTCAAACAATCTCCTGCAACAGTACGGGAGGTGTTCTGATGTCTGACTACCTGAAAGTGCTGCAAGCAATCAAAAGTTGCCCTAAGACTTTCCAGTCCAACTATGTACGGAACAATGCGAGCCTCGTAGCGGAGGCCGCTTCCCGTGGTCACATCTCGTGCCTGactacctg                                               >  1:22154/1‑298 (MQ=255)`  
                                                                                                                                                                                                                                                              `ggTAAACAGAAGGAAATGATTGTCCAGTTCCTGACACACGGTATGGAAGGATTGATGACATTCGTAGTACGTACATCATTTCGTGAGGCCATTAAGGACATGCACGAAGAGTATGCAGATAAGGACTCTTTCAAACAATCTCCTGCAACAGTACGGGAGGTGATCTGATGTCTGACTACCTGACAGTGCTGCAAGCAATCAAAAGTTGCCCTAAGACTTTCCAGTCACACTATGTACGGAACAATGCGAGCCGCGTAGCGGAGGCCGCTTCCCGTGGTCACATCTAGTGCATCACGACTAg                                               >  1:103407/1‑301 (MQ=255)`  
                                                                                                                                                                                                                                                              `ctattaccctatgctgactcagagtaaagaggacttggttgtatgGAAGGATTGATGACATTCGTAGTACGTACATCATTTCGTGAGGCCATTAAGGACATGCACGAAGAGTATGCAGATAAGGACTCTTTCAAACAGTCTCCTGCAACAGTAAGGGAGGTGTTCTGATGTCTGACTACCTGAAAGTGCTGCAAGCACTCAAAAGTTGCCCTACGACTTTCCAGTCCAACTATGTACGGAACAATGCGAGCCTCGTAGCGGAGGCCGCTTCCCGTGGTCACATCTCGTGCCtgtctacttg                                               >  1:143025‑M2/46‑299 (MQ=255)`  
                                                                                                                                                                                                                                                              `ctattaccctatgctgactcagagtaaagaggacttggttgtatgGAAGGATTGATGACATTCGTAGTACGTACATCATTTCGTGAGGCCATTAAGGACATGCACGAAGAGTATGCAGATAAGGACTCTTTCAAACAATCTCCTGCAACAGTACGGGAGGTGTTCTGATGTCTGACTACCTGAAAGTGCTGCAAGCAATCAAAAGTTGCCCTAAGACTTTCCAGTCCAACTATGTACGGAACAATGCGAGCCTCGTAGCGGAGGCcgcttcccgtggtcacatcctgtctcttatacccct                                               >  1:307444‑M2/46‑284 (MQ=255)`  
                                                                                                                                                                                                                                                              `aaagagacagatgctgactcagagtaaagaggacttggttgtatgGAAGGATTGATGACATTCGTAGTACGTACATCATTTCGTGAGGCCATTAAGGACATGCACGAAGAGTATGCAGATAAGGACTCTTTCAAACAATCTCCTGCAACAGTACGGGAGGTGTTCTGATGTCTGACTACCTGAAAGTGCTGCAAGCAATCAAAAGTTGCCCTAAGACTTTCCAGTCCAACTATGTACGGAACAATGCGAGCCTCGTAGCGGAGGCCGCTTCCCGTGGTCACATCTCGTGCCTGACTACTAg                                               <  1:25323‑M2/256‑1 (MQ=255)`  
                                                                                                                                                                                                                                                                `cagaccctatgctgactcagagtaaagaggacttggttgtatgGAAGGATTGATGACATTCGTAGTACGTACATCATTTCGTGAGGCCATTAAGGACATGCACGAAGAGTATGCAGATAAGGACTCTTTCAAACAATCTCCTGCAACAGTACGGGAGGTGTTCTGATGTCTGACTACCTGAAAGTGCTGCAAGCAATCAAAAGTTGCCCTAAGACTTTCCAGTCCAACTATGTACGGAACAATGCGAGCCTCGTAGCGGAGGCCGCTTCCCGTGGTCACATCTCGTGCCTGACTACTAGTg                                             <  1:93044‑M2/258‑1 (MQ=255)`  
                                                                                                                                                                                                                                                                `agatgtgtataagagacaggagtaaagaggacttggttgtatgGAAGGATTGATGACATTCGTAGTACGTACATCATTTCGTGAGGCCATTAAGGACATGCACGAAGAGTATGCAGATAAGGACTCTTTCAAACAATCTCCTGCAACAGTACGGGAGGTGTTCTGATGTCTGACTACCTGAAAGTGCTGCAAGCAATCAAAAGTTGCCCTAAGACTTTCCAGTCCAACTATGTACGGAACAATGCGAGCCTCGTAGCGGAGGCCGCTTCCCGTGGTCACATCTCGTGCCTGACTACTAGTg                                             <  1:83263‑M2/258‑1 (MQ=255)`  
                                                                                                                                                                                                                                                                `aaaaCAGAAGAAAATGATTGTCCAGTTCCTGAAACACGTTATGGAAGGATTGATGACATTCGTAGTACGTACATCATTTCGTGAGGCCATTAAGGACATGCACGATGAGTATGCAGATAAGGACTCTTTCAACCAATCTCCTGCAACAGTACGGGAGGTGTTCTGATGTCTGACTACCTGCAGGTGCTGCAAGCAATCAAAAGTTGCCCTAAGACTTTCCAGTCCAACTATGTACGGAACAATGCGAGCCTCGTAGCGGAGGCCGCTTCCCGTGGTCACATCTCGTGCCTGACTACTAGTg                                             <  1:100120/300‑1 (MQ=255)`  
                                                                                                                                                                                                                                                                   `accctatgctgactcagagtaaagaggacttggttgtatgGAAGGATTGATGACATTCGTAGTACGTACATCATTTCGTGAGGCCATTAAGGACATGCACGAAGAGTATGCAGATAAGGACTCTTTCAAACAATCTCCTGCAACAGTACGGGAGGTGTTCTGATGTCTGACTACCTGAAAGTGCTGCAAGCAATCAAAAGTTGCCCTAAGACTTTCCAGTCCAACTATGTACGGAACAATGCGAGCCTCGTAGCGGAGGCCGCTTCCCGTGGTCACATCTCGTGCCTGACTACTAGTGGAc                                          >  1:339272‑M2/41‑301 (MQ=255)`  
                                                                                                                                                                                                                                                                   `accctatgctgactcagagtaaagaggacttggttgtatgGAAGGATTGATGACATTCGTAGTACGTACATCATTTCGTGAGGCCATTAAGGACATGCACGAAGAGTATGCAGATAAGGACTCTTTCAAACAATCTCCTGCAACAGTACGGGAGGTGTTCTGATGTCTGACTACCTGAAAGTGCTGCAAGCAATCAAAAGTTGCCCTAAGACTTTCCAGTCCAACTATGTACGGAACAATGCGAGCCTCGTAGCGGAGGCCGCTTCCCGTGGTCACATCTCGTGCCTGACTACTAGTGGAc                                          >  1:262936‑M2/41‑301 (MQ=255)`  
                                                                                                                                                                                                                                                                        `atgctgactcagagtaaagaggacttggttgtatgGAAGGATTGATGACATTCGTAGTACGTACATCATTTCGTGAGGCCATTAAGGACATGCACGAAGAGTATGCAGATAAGGACTCTTTCAAACAATCTCCTGCAACAGTACGGGAGGTGTTCTGATGTCTGACTACCTGAAAGTGCTGCAAGCAATCAAAAGTTGCCCTAAGACTTTCCAGTCCAACTATGTACGGAACAATGCGAGCCTCGTAGCGGAGGCCGCTTCCCGTGGTCACATCTCGTGCCTGCctactagtgctgtctct                                     >  1:244985‑M2/36‑293 (MQ=255)`  
                                                                                                                                                                                                                                                                         `ggAAATGATTGTCCAGTTCCTGACACACGGTATGGAAGGATTGATGACATTCGTAGTACGTACATCATTTCGTGAGGCCATTAAGGACATGCACGAAGAGTATGCAGATAAGGACTCTTTCAAACAATCTCCTGCAACAGTACGGGAGGTGTTCTGATGTCTGACTACCTGAAAGTGCTGCAAGCAATCAAAAGTTGCCCTAAGACTTTCTAGTCCAACTATGTACGGAACAATGCGAGCCTCGTAGCGGAGGCCGCTTCCCGTGGTCACATCTCGTGCCTGACTACTAGTGGACGTAACg                                    <  1:300170/301‑1 (MQ=255)`  
                                                                                                                                                                                                                                                                          `tgggctcggagatgtgtataagagacaggTATGGAAGGATTGATGACATTCGTAGTACGTACATCATTTCGTGAGGCCATTAAGGACATGCACGAAGAGTATGCAGATAAGGACTCTTTCAAACAATCTCCTGCAACAGTACGGGAGGTGTTCTGATGTCTGACTACCTGAAAGTGCTGCAAGCAATCAAAAGTTGCCCTAAGACTTTCCAGTCCAACTATGTACGGAACAATGCGAGCCTCGTAGCGGAGGCCGCTTCCCGTGGTCACATCTCGTGCCTGACTACTAGTGGACGTAACgg                                   <  1:296536/274‑1 (MQ=255)`  
                                                                                                                                                                                                                                                                          `gtataagatacagaaagagtacttggttgtatgGAAGGATTGATGACATTCGTAGTACGTACATCATTTCGTTAGGCCATTAAGGACATGCACGAAGAGTATGCAGATAAGGACTCTTTCAAACAATCTCCTGCAACAGTACGGGATGTGTTCTGATGTCTGACTACCTGACAGTGCTGCAAGCAATCAAAAGTTGCCCTAAGACTTTCCAGTCCAACTATGTACGGAACAATGCGAGCCTCGTAGCGGAGGCCGCTTCCCGTGGTCACATCTCGTGCCTGACTACTAGTGGACGTAACgg                                   <  1:46108‑M2/268‑1 (MQ=255)`  
                                                                                                                                                                                                                                                                                     `gtaaagaggacttggttgtatgGAAGGTTTGATGACATTCGTAGTACGTACATCATTTAGTGAGGCCATTAAGGACATGCACGCAGAGTATGCAGATAAGGACTCTTTCAAACAATCTCCTGCAACATACCGGGAGGTGTTTTGATGTCTGACTACCTGAAAGTGCTGCAAGCAATCAAAAGTTGCCCTAAGACTTTCCAGTCTAACTATGTACGGAACTATGCTAGCTTCGTAGCGTAGGCCTCTTCACGTTGTCACATCACGTGCCTGACTTCTAGGGGACCTCACGGACGCGCTTggg                        >  1:24226‑M2/23‑301 (MQ=255)`  
                                                                                                                                                                                                                                                                                        `gTTCCTGACACACGGTATGGAAGGATTGATGACATTCGTAGTACGTACATCATTTCGTGAGGCCATTAAGGACATGCACGAAGAGTATGCAGATAAGGACTCTTTCAAACAATCTCCTGCAACAGTACGGGAGGTGTTCTGATGTCTGACTACCTGAAAGTGCTGCAAGCAATCAAAAGTTGCCCTAAGACTTTCCAGTCCGACTATGTACGGAACAATGCGAGCCTCGTAGCGGAGGCCGCTTCCCGTGGTCACATCTCGTGCCTGACTACTAGTGGACGTAACGGTGGCGCTTGGGaaa                     >  1:128318/1‑301 (MQ=255)`  
                                                                                                                                                                                                                                                                                          `aaggacttgggtgtatgGAAGGATTGATGACATTCGTAGTACGTACATCATTTCGTGAGGCCATTAAGGACATGCACGAAGAGTATGCAGATAAGGACTCTTTCAAACAATCTCCTGCAACAGTACGGGAGGTGTTCTGATGTCTGACTACCTGAAAGTGCTGCAAGCAATCAAAAGTTGCCCTAAGACTTTCCAGTCCAACTATGTACGGAACAATGCGAGCCTCGTAGCGGAGGCCGCTTCCCGTGGTCACATCTCGTGCCTGACTACTAGTGGACGTAACGGTGGCGCTTGGGAAATc                   <  1:18010‑M2/284‑1 (MQ=255)`  
                                                                                                                                                                                                                                                                                            `cttacacacGGTATGGAAGGATTGATGACATTCGTAGTACGTACATCATTTCGTGAGGCCATTAAGGACATGCACGAAGAGTATGCAGATAAGGACTCTTTCAAACAATCTCCTGCAACAGTACGGGAGGTGTTCTGATGTCTGACTACCTGAAAGTGCTGCAAGCAATCAAAAGTTGCCCTAAGACTTTCCAGTCCAACTATGTACGGAACAATGCGAGCCTCGTAGCGGAGGCCGCTTCCCGTGGTCACATCTCGTGCCTGACTACTAGTGGACGTAACGGTGGCGCTTGGGAAATCAc                 <  1:27893/298‑1 (MQ=255)`  
                                                                                                                                                                                                                                                                                                     `tgtatgGAAGGATTGATGACATTCGTAGTACGTACATCATTTCGTGAGGCCATTAAGGACATGCACGAAGAGTATGCAGATAAGGACTCTTTCAAACAATCTCCTGCAACAGTACGGGCGGTGTTCTGATGTCTGACTACCTGAAAGTGCTGCAAGCAATCAAAAGTTGCCCTAAGACTTTCCAGTCCAACTATGTACGGAACAATCCGAGCCTCGTAGCGGAGGCCGCTTCCCGTGGTCACATCTCGTGCCTGACTACTCGTGGACGTAACGGTGGCGCTTGGGAAATCACTGCTTCcgt        >  1:223793‑M2/7‑300 (MQ=255)`  
                                                                                                                                                                                                                                                                                                      `gtatgGAAGGATTGATGACATTCGTAGTACGTACATCATTTCGTGAGGCCATTAAGGACATGCACGAAGAGTATGCAGATAAGGACTCTTTCAAACAATCTCCTGCAACAGTACGGGAGGTGTTCTGATGTCTGACTACCTGAAAGTGCTGCAAGCAATCAAAAGTTGCCCTAAGACTTTCCAGTCCAACTATGTACGGAACAATGCGAGCCTCGTAGCGGAGGCCGCTTCCCGTGGTCACATCTCGTGCCTGACTACTAGTGGACGTAACGGTGGCGCTTGGGAAATCACTGCTTCCGGt       <  1:304023‑M2/296‑1 (MQ=255)`  

                                                                                                                                                                                                                                                                                                           `|`                                                                                                                                                                                                                                                                                                              
`GACATTCTTGCGGTTGTGGTTGACGAAGATGTGCTTATTTTCACATGGGACGAAGTAAAAGGTAAGACATCCATGTGTGTCGGCAAGAGAAACAAAGGCATAAAACTATAGGAGAAATTATTATGGCTATGACAAAGAAATTTAAAGTGTCCTTCGACGTTACCGCAAAGATGTCGTCTGACGTTCAGGCAATCTTAGAGAAAGATATGCTGCATCTATGTAAGCAGGTCGGCTCAGGTGCGATTGTCCCCAATGGTAAACAGAAGGAAATGATTGTCCAGTTCCTGACACACGGTATGGAAGGATTGATGACATTCGTAGTACGTACATCATTTCGTGAGGCCATTAAGGACATGCACGAAGAGTATGCAGATAAGGACTCTTTCAAACAATCTCCTGCAACAGTACGGGAGGTGTTCTGATGTCTGACTACCTGAAAGTGCTGCAAGCAATCAAAAGTTGCCCTAAGACTTTCCAGTCCAACTATGTACGGAACAATGCGAGCCTCGTAGCGGAGGCCGCTTCCCGTGGTCACATCTCGTGCCTGACTACTAGTGGACGTAACGGTGGCGCTTGGGAAATCACTGCTTCCGGTACTCG  >  NC_001604.1/16729‑17328`

  

Alignment Legend

Aligned base mismatch/match (shaded by quality score): `ATCG/ATCG` `< 3 ≤` `ATCG/ATCG` `< 11 ≤` `ATCG/ATCG` `< 23 ≤` `ATCG/ATCG` `< 34 ≤` `ATCG/ATCG` `< 38 ≤` `ATCG/ATCG`

Unaligned base: `atcg`    Masked matching base: `atcg`    Alignment gap: `‑`    Deleted base: `‑`

Reads not counted as support for junction

read\_name Not counted due to insufficient overlap past the breakpoint.

read\_name Not counted due to not crossing MOB target site duplication.
